# Cirsoid Aneurysm: Report of Two Cases With Cosmetic Surgical Treatment and Narrative Review of the Literature

**DOI:** 10.1155/cris/9907175

**Published:** 2026-10-07

**Authors:** Henrique Salles Barbosa, Renata Salles Barbosa, Aline Cristina Pavani, Mariana Paes Levasseur, Mariana Fernandes Carvalho, João Frederico Vitorino Coelho Chaves, Luís Guilherme Delage da Fonseca

**Affiliations:** ^1^ Department of Surgery Medicine School Federal University of Juiz de Fora Juiz de Fora Minas Gerais Brazil ufjf.br; ^2^ Department of Surgery Medicine School University President Antônio Carlos of Juiz de Fora Juiz de Fora Minas Gerais Brazil; ^3^ Department of Anesthesiology Hospital House of Holy Mercy of Juiz de Fora Juiz de Fora Minas Gerais Brazil; ^4^ Department of Surgery Hospital House of Holy Mercy of Juiz de Fora Juiz de Fora Minas Gerais Brazil

**Keywords:** arteriovenous fistula, arteriovenous malformation, cirsoid aneurysm, vascular malformation

## Abstract

Cirsoid aneurysm (CA) is a rare high‐flow arteriovenous malformation (AVM) of the scalp that may cause cosmetic deformity, neurologic symptoms, skin ulceration, or hemorrhage. We report two men with left frontoparietal lesions supplied by the frontal branch of the superficial temporal artery. A 37‐year‐old man presented with a progressively enlarging pulsatile mass of 3 years’ duration. Duplex ultrasonography and computed tomography angiography (CTA) demonstrated a high‐flow scalp AVM with extensive venous drainage and no intracranial communication. A 27‐year‐old man presented with a 1‐year history of a pulsatile mass that underwent rapid enlargement, ulceration, and massive bleeding. Temporary hemostasis was achieved by compression, and CTA showed a similar lesion without intracranial communication. Both patients underwent complete surgical excision through a left coronal incision without preoperative embolization. The feeding artery was ligated, the abnormal venous network was resected, and the ulcerated skin was excised in the second patient. Neither procedure required transfusion, and both postoperative courses were uneventful. At clinical follow‐up of 6 months in the first patient and 3 months in the second, facial muscle function was preserved, cosmetic outcomes were satisfactory, and no clinical recurrence was detected. These cases support surgical excision as an effective option for localized, surgically accessible CAs with a limited arterial supply, while treatment should be individualized according to lesion extent, vascular anatomy, symptoms, hemorrhagic risk, and cosmetic objectives.

## 1. Introduction

Cirsoid aneurysm (CA), also termed a scalp arteriovenous fistula or arteriovenous malformation (AVM), is a rare high‐flow vascular lesion composed of direct communications between feeding arteries and draining scalp veins without an intervening capillary bed. Despite the historical term “aneurysm,” it is not a true arterial aneurysm[1–4].

Lesions may be congenital or acquired after trauma, surgery, inflammation, infection, or other iatrogenic injury. Branches of the external carotid artery—most often the superficial temporal, occipital, and posterior auricular arteries—commonly supply the lesion. Clinical manifestations range from a pulsatile mass, bruit, thrill, headache, tinnitus, and cosmetic deformity to skin ulceration, hemorrhage, and, rarely, heart failure [1, 3, 5–8].

Duplex ultrasonography, computed tomography angiography (CTA), magnetic resonance angiography (MRA), and digital subtraction angiography (DSA) can define vascular anatomy and assess intracranial communication. Treatment options include surgical excision, endovascular or percutaneous embolization, and combined approaches. Because CAs are uncommon and anatomically heterogeneous, management should be individualized according to lesion extent, feeding and draining vessels, symptoms, hemorrhagic risk, surgical accessibility, and cosmetic goals [1, 8–10].

We report two CAs supplied solely by the frontal branch of the superficial temporal artery and treated by surgical excision through a coronal incision with favorable clinical, functional, and cosmetic outcomes.

## 2. Case Presentation

We retrospectively reviewed two patients with CAs treated by surgical excision through a coronal incision. Demographic, clinical, imaging, operative, and postoperative follow‐up data were collected. Written informed consent for publication of the cases and accompanying images was obtained from both patients.

In both patients, clinical suspicion prompted duplex ultrasonography, followed by arterial CTA of the neck and skull to characterize the lesion, guide surgical planning, and assess for intracranial communication.

### 2.1. Case 1

A 37‐year‐old Brazilian man without comorbidities presented with a 3‐year history of a progressively enlarging pulsatile mass in the left frontoparietal region (Figure 1). The patient denied any history of trauma or prior surgical intervention. No other relevant personal or family medical history was reported.

**Figure 1:**
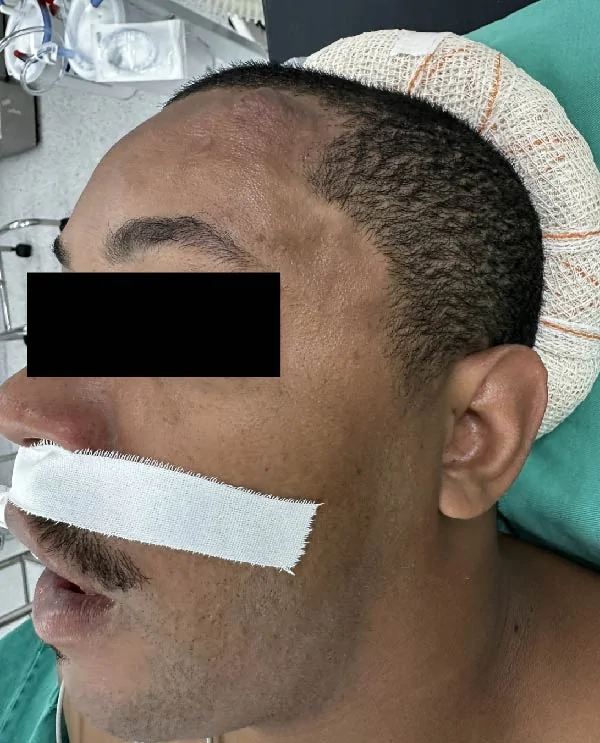
(Case 1): Clinical appearance of a cirsoid aneurysm with an approximately 3‐year history.

Arterial Doppler findings were consistent with a high‐flow AVM. CTA was performed to complement the diagnostic evaluation. CTA revealed a left frontoparietal CA supplied by the frontal branch of the left superficial temporal artery, with extensive scalp venous drainage (Figure 2).

**Figure 2:**
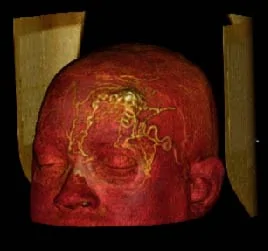
(Case 1): Preoperative CTA demonstrating the cirsoid aneurysm with volumetric reconstruction.

Surgical treatment was performed under general anesthesia through a left coronal incision (Figure 3). After elevation of the scalp flap and identification and control of the feeding artery, the arteriovenous fistula and its main draining veins were completely excised (Figure 4).

**Figure 3:**
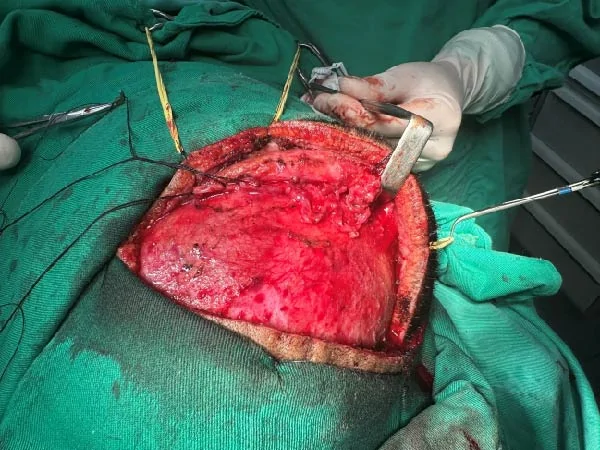
(Case 1): Intraoperative view after left coronal incision showing dissection of the feeding artery and draining venous plexus.

**Figure 4:**
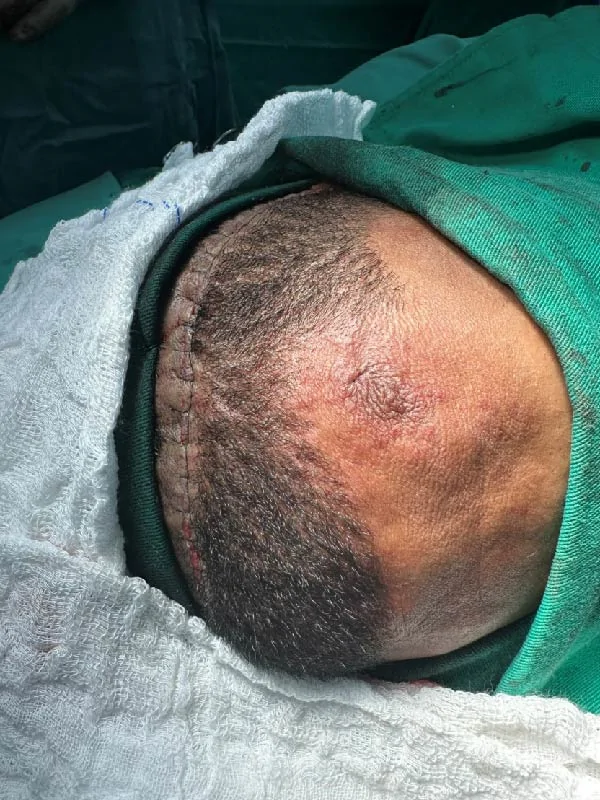
(Case 1): Final appearance after coronal incision surgery.

The postoperative course was uneventful, and the patient was discharged on the second postoperative day. Follow‐up evaluation demonstrated complete resolution of the AVM, with intact facial muscle function and satisfactory cosmetic outcome (Figures 5 and 6).

**Figure 5:**
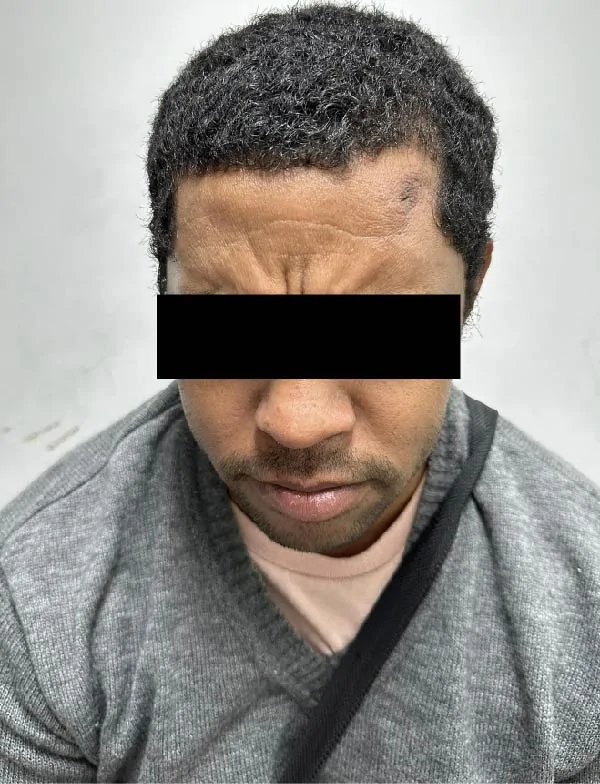
(Case 1): Three‐month postoperative follow‐up showing cosmetic outcome and intact facial muscle function.

**Figure 6:**
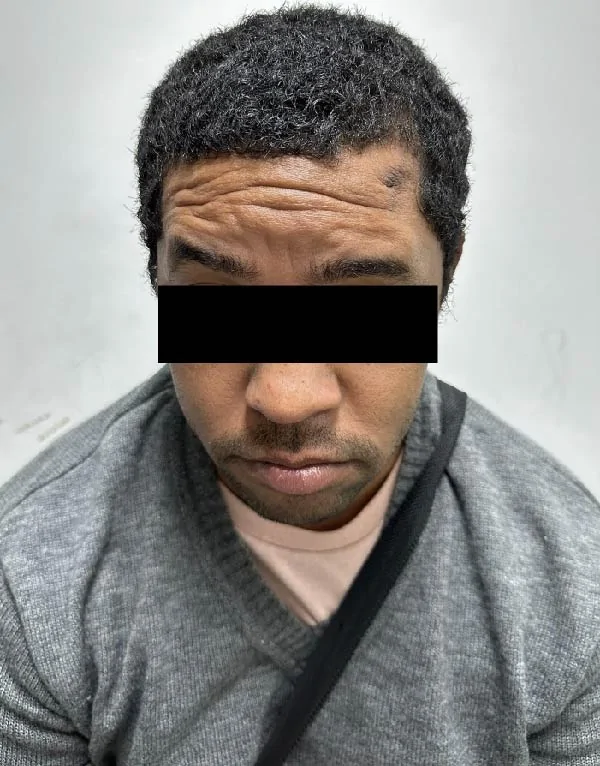
(Case 1): Three‐month postoperative follow‐up showing adequate wound healing and unchanged facial muscle function.

### 2.2. Case 2

A 27‐year‐old Brazilian man without comorbidities presented with a pulsatile mass in the left frontoparietal region, evolving over 1 year with recent sudden enlargement. He was being followed up as an outpatient while awaiting surgical planning. Prior to the scheduled intervention, the CA showed rapid expansion with skin ulceration and an episode of massive bleeding (Figure 7).

**Figure 7:**
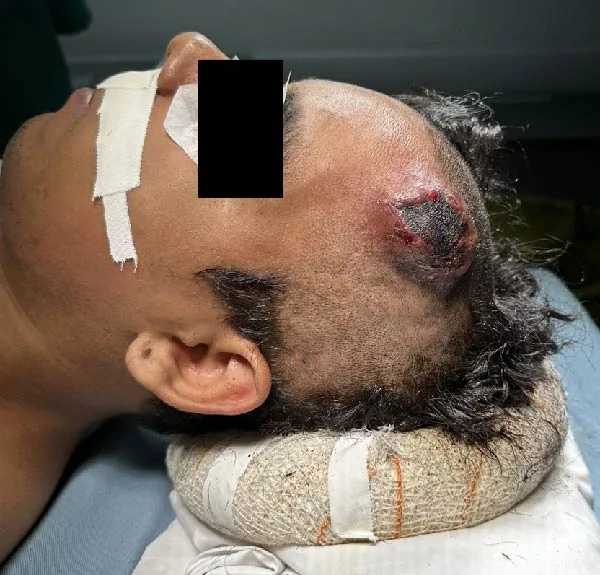
(Case 2): Large cirsoid aneurysm with skin ulceration and adherent blood clots.

In the emergency room, temporary hemostasis was achieved using a compression dressing. Subsequently, CTA was performed to investigate intracranial communications, which were not identified (Figure 8). Surgery was scheduled for the following day. Surgical excision was performed through a left coronal incision, extended across the scalp, crossing the midline, and dissected up to the superciliary region (Figure 9).

**Figure 8:**
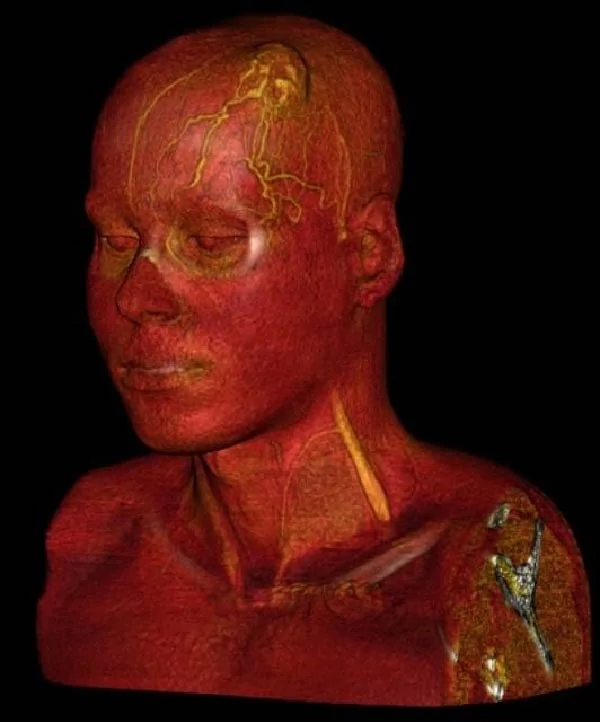
(Case 2): Preoperative CTA with volumetric reconstruction showing a large cirsoid aneurysm.

**Figure 9:**
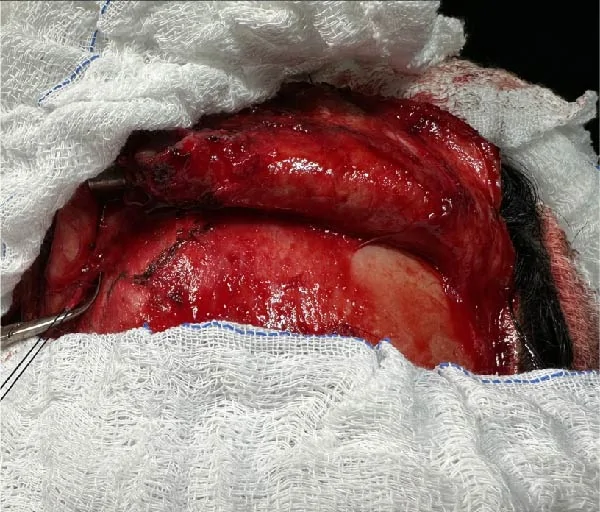
(Case 2): Extension of the left coronal incision to expose the cirsoid aneurysm.

Exposure through the coronal incision allowed easy identification and ligation of the single feeding artery (the frontal branch of the superficial temporal artery) and resection of the venous network associated with the arteriovenous fistula. Excision of the area of cutaneous ischemia was required, with adequate intraoperative approximation of the wound edges (Figure 10).

**Figure 10:**
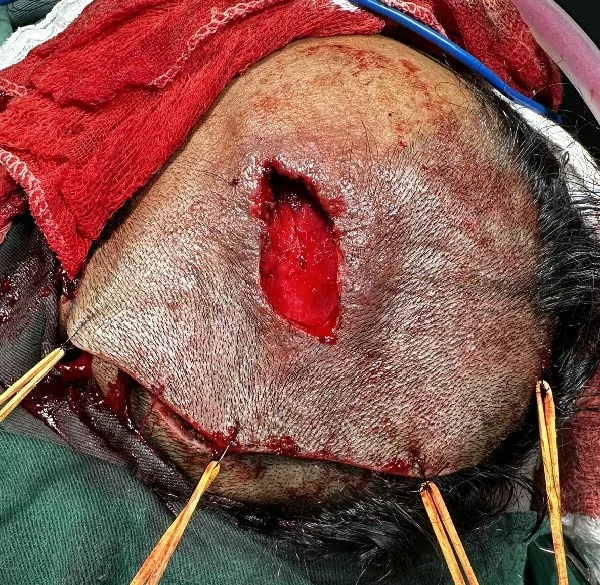
(Case 2): Intraoperative view of the scalp flap following resection of the cirsoid aneurysm and excision of the ulcerated area.

Postoperative recovery was uneventful, and the patient was discharged on the forth postoperative day. Clinical follow‐up showed satisfactory postoperative evolution, with a satisfactory cosmetic scar and complete preservation of facial muscle function (Figures 11 and 12).

**Figure 11:**
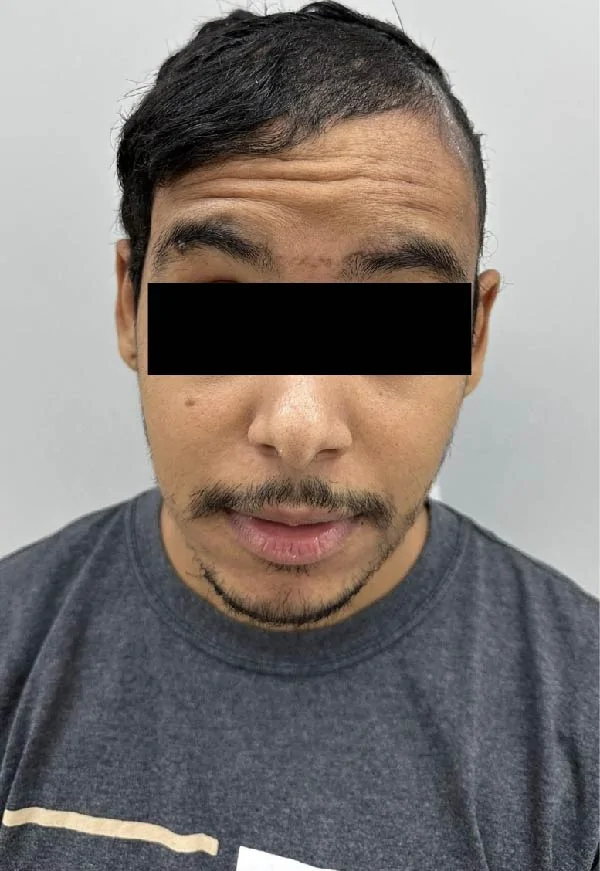
(Case 2): Postoperative appearance at 1 month. Cosmetic result with preservation of facial mimic muscles.

**Figure 12:**
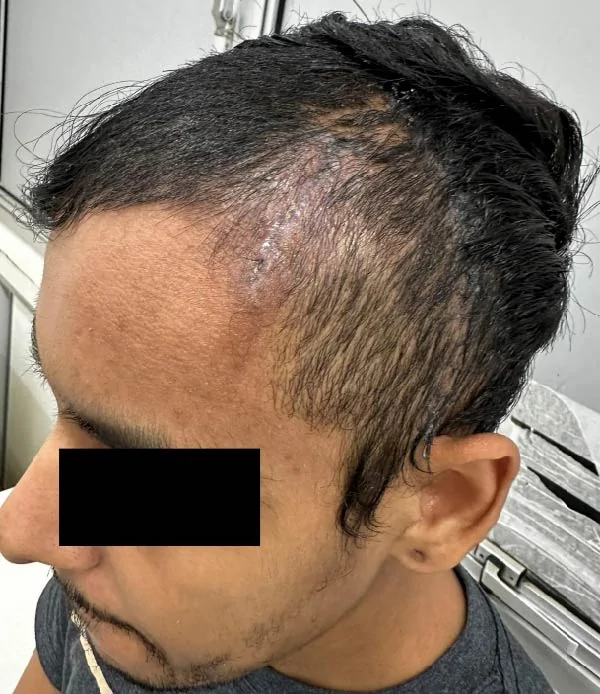
(Case 2): Postoperative appearance at 1 month. Cosmetic outcome with a satisfactory cosmetic scar.

## 3. Discussion

CAs are rare subcutaneous AVMs that occur on the scalp [1]. They account for ~8% of AVMs [8]. In fact, despite the nomenclature, they are arteriovenous fistulas between feeding arteries and draining veins rather than true aneurysms [1]. CAs may occur in the periosteal layer beneath the galea aponeurotica, within the temporal fascia, or in the epicranial aponeurosis [4]. Its distribution is evenly divided among the frontal, temporal, and parietal regions [4].

Regarding etiology, CAs may arise spontaneously or result from trauma (including neurosurgical manipulation, temporomandibular joint manipulation, or hair transplantation) [4], as well as iatrogenic, inflammatory, or congenital causes [3, 11]. Alghamdi et al. [9] reported that ~25% of CAs were congenital, 19.8% traumatic, and 14.3% iatrogenic. On the other hand, Elkiran et al. [12] reported that while most CAs are congenital, 10%–55% of cases arise after trauma.

Congenital CAs may initially be mild but develop more aggressively during puberty and adolescence [8]. They usually present with symptoms in the third decade of life, with a higher prevalence in men [4]. Proposed causes of congenital CAs include persistence of primitive arteriovenous fistulas, vascular hamartomas, and fistulous formations at sites of arteriovenous crossing [8].

It is hypothesized that many CAs originate from quiescent or nonpatent arteriovenous fistulas, which develop and become clinically relevant after local trauma [4, 12]. Arteriovenous fistulas may enlarge through the formation of new fistulous channels induced by vascular trauma [5]. Somatic mutations in the MAP2K1 gene can also lead to endothelial dysfunction and the development of CAs [5].

In the pathophysiology of CAs, there are two accepted theories. They may originate either from direct trauma to the artery and vein (laceration theory) or from rapid proliferation of endothelial cells in damaged vasa vasorum, resulting in angiogenesis (disruption theory) [7, 13, 14]. In these lesions, endothelial vascular growth factors drive continued growth, endothelial proliferation, angiogenesis, and abnormal vascular connections [7, 13].

Yokouchi et al. [13, 15] proposed a classification of CAs into three types. In type A, a single fistula is supplied by one proximal artery; in type B, a single fistula is supplied by multiple proximal arteries; and in type C, multiple fistulas are associated with plexiform feeding arteries and dilated draining veins [13].

In addition to Yokouchi’s classification, Shenoy proposed a division into two groups. Group I includes primary scalp vascular malformations supplied by branches of the external carotid, vertebral, or ophthalmic arteries. Group II consists of venous dilations of the scalp with intracranial connections [13, 16].

The arteriovenous lesions that constitute CAs may initially present as pulsatile subcutaneous masses, sometimes misdiagnosed as benign scalp irregularities [4]. These masses, initially small (as in Case 1), can enlarge to deforming dimensions and evolve into large vascular lesions (as in Case 2) [3, 4, 17].

The most common signs and symptoms of CAs include a pulsatile mass, audible bruit, palpable thrill, tinnitus, paresthesia, headache, local pain, seizures, bleeding, ulceration, and, in rare cases, heart failure [1, 4, 8, 10, 14, 16, 18]. The classic clinical triad of CA consists of a mass with local growth, pain, and a thrill that disappears with manual compression of the feeding artery [7]. Some CAs present as nonpulsatile lesions, although these are less common [14]. The pulsatile mass commonly has a significant cosmetic and psychosocial impact on patients, in addition to the clinical signs and symptoms [5, 8].

In our two reported cases, a pulsatile mass with a characteristic thrill was present, which ceased immediately with compression of the superficial temporal artery. None of the patients reported local pain, headache, or other symptoms. Cosmetic discomfort, however, was common in both cases and was the reason for seeking medical attention. Furthermore, in the second case, sudden enlargement and rupture of the CA, with massive bleeding, required surgical intervention.

The superficial temporal artery is the most frequently involved feeding vessel in CAs. Because of its long and superficial course over the skull, it is more susceptible to trauma [3, 8, 10, 14]. Common additional feeders are the occipital, posterior auricular, and internal maxillary artery branches. Branches of the ophthalmic artery, which arise from the internal carotid artery, may also supply CAs [8].

They may be supplied by a single artery or by multiple arteries, with up to seven feeding vessels reported [7]. In our two cases, the frontal branch of the left superficial temporal artery was the sole feeding vessel of the CAs.

Differential diagnoses for CAs include aneurysm, venous or venolymphatic malformations, hematoma, lipoma, cyst, abscess, lymphadenopathy, and tumors. Clinically, CAs can be mistaken for low‐flow venous malformations such as cavernous hemangiomas and *sinus pericranii* [3, 14]. However, such low‐flow lesions lack an arteriovenous fistula and, therefore, do not demonstrate “flow void” signs on MRI [7].

Diagnostic imaging modalities for evaluating CAs include duplex ultrasonography, MRA, CTA, and DSA [1, 2, 9, 16, 19, 20].

Duplex ultrasonography is a valuable tool in the diagnosis of arteriovenous fistulas and CAs. Diagnostic criteria include identification of a feeding artery with low resistance, high‐velocity flow, and an arterial waveform pattern in the draining vein. The arteriovenous junction should also demonstrate turbulent, high‐velocity flow [19].

DSA remains the reference standard for defining feeding arteries, draining veins, and intracranial involvement and permits simultaneous endovascular treatment when indicated [10, 19, 21]. In the present cases, DSA was not performed because duplex ultrasonography and CTA consistently demonstrated a localized lesion supplied by a single superficial temporal artery branch, excluded intracranial communication, and provided sufficient information for open surgical planning; no endovascular procedure was planned.

MRA and CTA provide detailed information on the location, extent, and configuration of CAs [4]. CTA provides greater detail of the vascular anatomy than MRA. On MRA, CAs appear as multiple scalp “flow voids,” a characteristic finding [13]. According to several authors, CTA provides diagnostic accuracy comparable to DSA but with significantly lower risks [1, 9, 12].

Treatment is generally indicated for cosmetic deformity or psychosocial distress, disabling symptoms, progressive enlargement, skin compromise, or hemorrhagic risk. Management is challenging because of lesion location, complex vascularization, high‐flow dynamics, and cosmetic implications [1, 8, 9]. Surgical excision is a reference strategy when complete removal is feasible and may provide rapid symptom resolution, low recurrence, cost effectiveness, and favorable cosmetic outcomes [1, 8, 10, 14–16, 18]. However, treatment should be individualized by a multidisciplinary team according to size, location, vascular architecture, accessibility, and patient priorities [8, 9].

Reported surgical complications include intraoperative hemorrhage, lesion recurrence—often related to incomplete nidus removal—wound infection, scalp flap ischemia or necrosis, alopecia, and injury to the temporofrontal branch of the facial nerve with partial motor deficit [7–9, 14, 22]. Careful incision placement, flap elevation, vascular control, and identification of the facial nerve plane are therefore important.

Preoperative embolization may reduce flow and facilitate resection in extensive lesions, lesions with multiple feeders, or anatomically difficult lesions, and some authors have reported a risk of uncontrolled bleeding when surgery is performed without it [14, 21]. In our cases, surgery without embolization was selected because CTA demonstrated a localized, surgically accessible lesion with a single readily controlled feeding artery and no intracranial communication. Case 1 was treated primarily for progressive growth and cosmetic concern; case 2 required definitive treatment after rapid enlargement, ulceration, and hemorrhage. The anticipated benefit of preoperative embolization was therefore judged limited. Blood loss was minimal in both procedures, with no hemodynamic consequences or transfusion.

The optimal surgical approach to CAs consists of creating a scalp flap [13]. The location of the lesion and its feeding arteries guide the procedure[13]. A coronal or bicoronal incision provides straightforward access to anterior frontal and midline lesions [13]. A horseshoe incision provides effective access for excision of parietal or occipital lesions [13]. After incision and scalp flap elevation, the feeding artery can be readily dissected, ligated, and divided. Dissection can then be carried to the venous end of the CA, which should likewise be dissected, ligated, and divided [13].

In our two cases, a coronal incision placed behind the hairline allowed adequate exposure of the AVM. In the first case, bipolar cautery was used for hemostasis. In the second case, however, hemostasis was achieved with monopolar cautery and ligatures, and no major technical difficulties were encountered. After incision and scalp elevation, we employed the technique of folding the scalp over itself with the aid of self‐retaining retractors. This maneuver reduced flow in the small arteries supplying the scalp, minimizing bleeding during dissection.

Another treatment option for CAs is endovascular intervention [14]. It can be used alone or in combination with surgery for preoperative embolization and reduction of intraoperative bleeding [7, 9, 15, 16]. Embolization can be performed via transarterial, transvenous, or combined routes [1]. Direct puncture of the lesion and percutaneous embolization have also been described [23].

Endovascular techniques offer a less invasive and faster alternative to surgery. However, they carry inherent limitations and potential complications [1]. The risk of recurrence is increased, particularly when all feeding vessels are not adequately treated. Additionally, there is an increased risk of scalp pain, hair loss, necrosis over the lesion, hyperemia, and inadvertent embolization to other sites [1, 3, 15].

Endovascular intervention can reduce lesion volume and mitigate bleeding related to lesion size [1, 8, 18] In selected patients—particularly those with extensive lesions, difficult surgical access, or a high anticipated morbidity from open exposure—modern embolization techniques may offer a less invasive approach and favorable cosmetic results [6, 8, 9, 23] Conversely, incomplete occlusion may leave a residual mass or lead to recurrence, and embolization can cause pain, alopecia, skin necrosis, hyperemia, or nontarget embolization [1, 3, 15] Thus, embolization may be definitive in selected anatomies, used before surgery, or combined with resection; no single modality is uniformly superior for all CAs.

With the evolution of endovascular devices, technologies, and techniques, significant advances have been achieved in the treatment of complex vascular lesions of the head and neck [23]. However, CAs remain challenging for endovascular management due to their superficial location and marked vascular tortuosity [23].

A literature review reported a significantly higher complication rate for endovascular treatment compared with surgery (55.8% vs. 9.9%) [14]. However, reported outcomes vary, and several authors have suggested that endovascular treatment carries a relatively low risk of complications [6].

According to Sofela et al. [14], treatment techniques can be classified based on the size and number of feeding arteries and draining veins. Lesions exceeding 4 cm in diameter typically require a combined endovascular and surgical approach. Lesions smaller than 4 cm but supplied by multiple feeding arteries can be excised primarily or in combination with endovascular treatment. Lesions smaller than 4 cm, with few feeding arteries and draining veins, can be treated surgically or by endovascular embolization [13].

Clinical follow‐up was 6 months for Case 1 and 3 months for Case 2. Neither patient underwent routine postoperative imaging; at the latest assessment, both had complete clinical resolution, preserved facial muscle function, satisfactory cosmetic outcomes, and no clinical evidence of recurrence. Given the risk of residual disease and reported late recurrence, longer clinical surveillance should be considered, with imaging if symptoms or pulsatility recur [8, 9, 12].

This report is limited by its retrospective design, the inclusion of only two patients, the absence of routine imaging during follow‐up, and the short follow‐up duration. These factors restrict generalizability and preclude robust comparison of recurrence and complication rates between treatment modalities. Larger series with standardized imaging and longer surveillance are needed.

In conclusion, surgical excision can provide complete lesion removal with good functional and cosmetic outcomes in localized, accessible CAs with limited arterial supply [1, 3, 12, 14]. Endovascular or combined treatment remains valuable for selected complex lesions, and the strategy should be tailored to vascular anatomy, clinical urgency, hemorrhagic risk, and cosmetic goals [8, 9, 14].

## Funding

The authors received no financial support for the research, authorship, and/or publication of this article.

## Disclosure

All authors reviewed and approved the final version of the manuscript.

## Ethics Statement

The study was conducted in accordance with the Declaration of Helsinki, and the Ethics Committee of Participant Institution approved the research (Comitê de Ética em Pesquisa da Santa Casa de Misericórdia de Juiz de Fora). The number of approval is 92383325.4.0000.5139.

## Consent

Written informed consent to publish was obtained from the patients in the present study and the accompanying images.

## Conflicts of Interest

The authors declare no conflicts of interest.

## Data Availability

The data that support the findings of this study are available from the corresponding author upon reasonable request.
